# Tracking the time course of pathological patterns of lung injury in severe COVID-19

**DOI:** 10.1186/s12931-021-01628-9

**Published:** 2021-01-29

**Authors:** Thais Mauad, Amaro Nunes Duarte-Neto, Luiz Fernando Ferraz da Silva, Ellen Pierre de Oliveira, Jose Mara de Brito, Ellen Caroline Toledo do Nascimento, Renata Aparecida de Almeida Monteiro, Juliana Carvalho Ferreira, Carlos Roberto Ribeiro de Carvalho, Paulo Hilário do Nascimento Saldiva, Marisa Dolhnikoff

**Affiliations:** 1 Departamento de Patologia, Faculdade de Medicina da Universidade de São Paulo, Av. Dr. Arnaldo, 455, sala 1155, Cerqueira Cesar, São Paulo, Brazil; 2 Serviço de Verificação de Óbitos da Capital, Universidade de São Paulo, São Paulo, Brazil; 3 Departamento de Cardiopneumologia, Instituto Do Coração, Faculdade de Medicina da Universidade de São Paulo, São Paulo, Brazil

**Keywords:** COVID-19, Lung pathology, Minimally invasive autopsy, Diffuse alveolar damage, Pulmonary thrombosis

## Abstract

**Background:**

Pulmonary involvement in COVID-19 is characterized pathologically by diffuse alveolar damage (DAD) and thrombosis, leading to the clinical picture of Acute Respiratory Distress Syndrome. The direct action of SARS-CoV-2 in lung cells and the dysregulated immuno-coagulative pathways activated in ARDS influence pulmonary involvement in severe COVID, that might be modulated by disease duration and individual factors. In this study we assessed the proportions of different lung pathology patterns in severe COVID-19 patients along the disease evolution and individual characteristics.

**Methods:**

We analysed lung tissue from 41 COVID-19 patients that died in the period March–June 2020 and were submitted to a minimally invasive autopsy. Eight pulmonary regions were sampled. Pulmonary pathologists analysed the H&E stained slides, performing semiquantitative scores on the following parameters: exudative, intermediate or advanced DAD, bronchopneumonia, alveolar haemorrhage, infarct (%), arteriolar (number) or capillary thrombosis (yes/no). Histopathological data were correlated with demographic-clinical variables and periods of symptoms-hospital stay.

**Results:**

Patient´s age varied from 22 to 88 years (18f/23 m), with hospital admission varying from 0 to 40 days. All patients had different proportions of DAD in their biopsies. Ninety percent of the patients presented pulmonary microthrombosis. The proportion of exudative DAD was higher in the period 0–8 days of hospital admission till death, whereas advanced DAD was higher after 17 days of hospital admission. In the group of patients that died within eight days of hospital admission, elderly patients had less proportion of the exudative pattern and increased proportions of the intermediate patterns. Obese patients had lower proportion of advanced DAD pattern in their biopsies, and lower than patients with overweight. Clustering analysis showed that patterns of vascular lesions (microthrombosis, infarction) clustered together, but not the other patterns. The vascular pattern was not influenced by demographic or clinical parameters, including time of disease progression.

**Conclusion:**

Patients with severe COVID-19 present different proportions of DAD patterns over time, with advanced DAD being more prevalent after 17 days, which seems to be influenced by age and weight. Vascular involvement is present in a large proportion of patients, occurs early in disease progression, and does not change over time.

## Introduction

Several autopsy studies have described lung pathology in patients that died due to SARS-CoV-2 infection, showing that most of the patients presented different stages of diffuse alveolar damage (DAD) and a high frequency of macro and microvascular thrombosis, leading to the clinical picture of Acute Respiratory Distress Syndrome (ARDS) [1–3]. The direct action of SARS-CoV-2 in lung cells and the dysregulated immuno-coagulative pathways activated in ARDS may influence patterns and extension of pulmonary involvement in severe COVID-19, that are modulated by disease duration and individual factors.

ARDS is a severe pulmonary condition caused by different noxious stimuli, which have in common the capacity of damaging the alveolar-capillary barrier. The alteration of permeability promoted by the aggression to the air/blood interface causes significant exudation of plasma components and inflammatory cells into alveolar space, altering gas diffusion and disrupting alveolar stability by surfactant dysfunction. If injury progresses, the inflammatory condition elicits a progressive organization of the intra-alveolar exudate and permanent fibrosis and distortion of the distal pulmonary parenchyma [4]. Autopsy studies confirmed that pulmonary fibrosis is not an uncommon finding in COVID-19 and anti-fibrotic therapeutic measures have been proposed to minimize pulmonary fibrotic remodelling in affected patients [5–8]. Polak et al. performed a systematic review of 69 COVID-19 autopsies, showing changes compatible with acute DAD, fibroproliferative DAD and vascular pathology in the lungs. Such patterns seemed to occur simultaneously in the lungs along time, but with a predominance of the fibrotic pattern after three weeks of disease [9].

COVID-19 is also associated with pulmonary thrombotic events that may be observed in different segments of pulmonary circulation, from capillaries to the large arterial branches. The vascular thrombotic events may significantly contribute to the respiratory insufficiency of COVID-19 [3, 10]. There is a lot of ongoing discussion on the pathogenic mechanisms of the vascular disease in COVID-19. Whereas endothelial dysfunction in COVID-19 is certainly caused by disruption of coagulation and fibrinolytic pathways associated with ARDS, Sars-CoV-2 directly infects the endothelial cells [11]. It is possible that a multitude of sequential injuries to endothelium occurs in COVID-19, leading to the disproportional larger number of systemic and pulmonary thrombotic events when compared to other causes of ARDS [12]. A predominance of direct effects of infection by the virus would cause immediate disease, whereas the indirect effects of endothelial activation linked to the development of ARDS would theoretically appear after some time of disease evolution.

COVID-19 significantly impacted the Brazilian population and the State of São Paulo, the region with the highest number of cases in the country. The São Paulo University Academical Hospital is the reference centre for the treatment of severe cases of COVID-19 in São Paulo and responded to such challenges by allocating 900 hospital beds (300 intensive care beds) exclusively dedicated to COVID-19 patients from March to August 2020. We investigated the pathology of severe fatal COVID-19 by implementing a protocol of ultrasound-guided minimally invasive autopsies (MIA/US) which was conceived to reduce the risk of obtaining post-mortem tissue samples in a disease with high contagiousness [3].

In this study, we present the results of a quantitative histopathological study designed to describe the proportions of different lung pathology patterns in severe COVID-19 patients along the disease evolution, with emphasis on its vascular and fibrotic alterations. We further attempted to identify whether demographic-clinical characteristics would influence the frequency/distribution of histopathological patterns.

## Methods

### Population

This study was approved by the National Ethical Committee Board (CAAE # 3 30364720.0.0000.0068), and autopsies were performed after written informed consent by the next-of-kin. The procedures were performed at the Image Platform in the Autopsy Room, a research centre in the University of Sao Paulo Medical School, located next to the Autopsy Service of Sao Paulo University (https://pisa.hc.fm.usp.br/). During the study period there were 4507 admissions due to COVID-19 in the hospitals linked to the Medical School, with 1184 deaths. Our group performed 54 MIA/US in patients suspected or confirmed of having COVID-19 during the period. For this study, we included 41 adult patients that died due to confirmed acute pulmonary disease related to COVID-19.

### Autopsy protocol

MIA/US protocol was described previously [13] and was adapted for COVID-19 for safety reasons [3]. Briefly, bodies were kept in the supine position and lung tissue was sampled from eight regions, in a combination of upper and lower chest (lateral and medial, 4 sites) and right and left lungs (2 sides). In each sampling site, six samples were collected. Such protocol resulted in 48 tissue samples collected from eight pulmonary regions for each case. Immediately after sampling, pulmonary samples were immersed in 10% formalin solution for 24 h and subsequently processed for routine paraffin embedding and stained with H&E. The researchers that performed MIA/US did not participate in the histopathological evaluation.

### Histopathological analysis

**Fig. 1:**
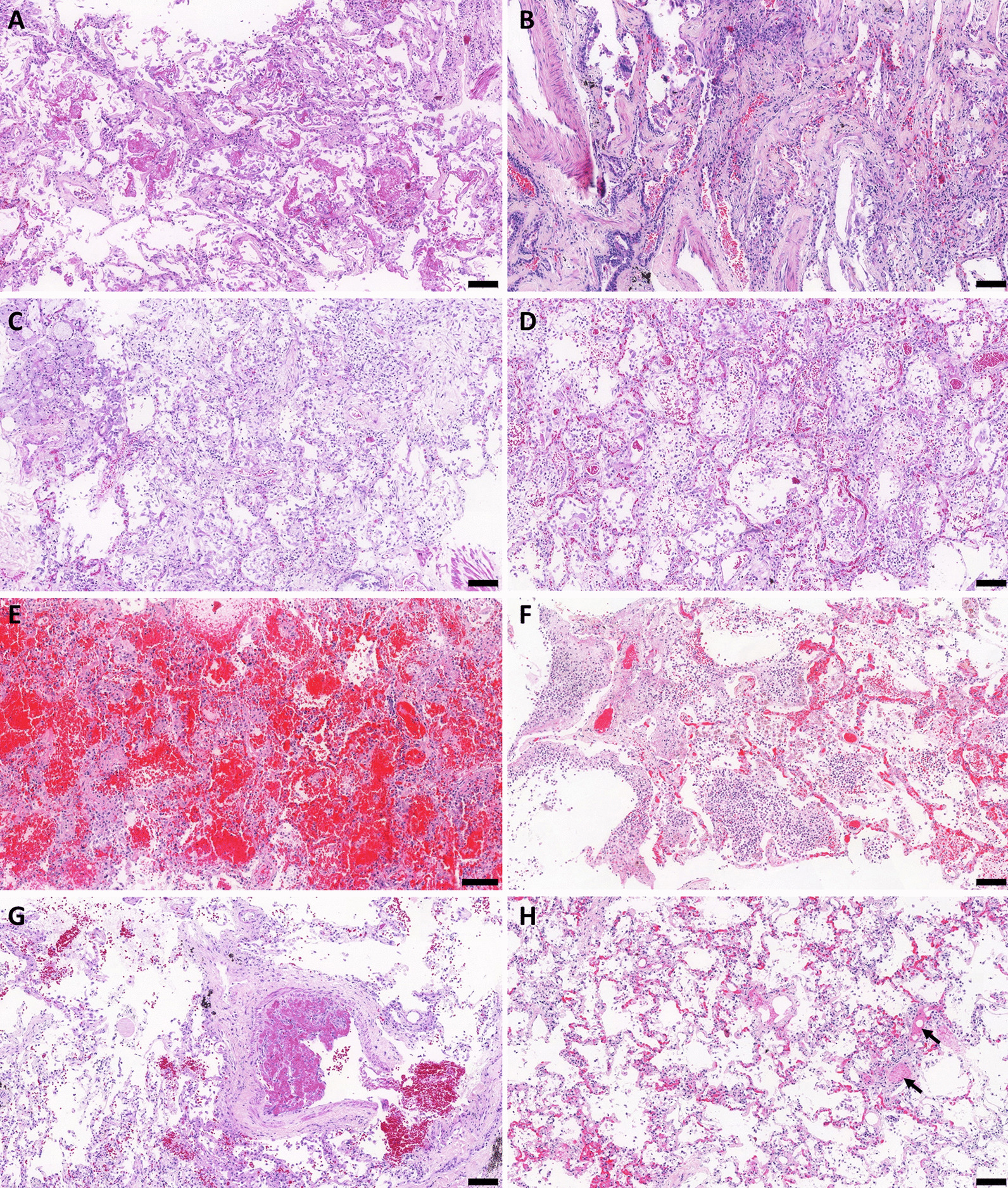
Histopathological parameters. **a** Diffuse Alveolar Damage, exudative. Observe the septa with mixed inflammatory infiltrate, hyaline membranes, desquamated pneumocytes and macrophages in the alveolar lumen. **b** Diffuse Alveolar Damage, advanced. Pulmonary architecture is altered, with fibrotic bundles substituting the alveolar parenchyma and distortion of the wall of an airway. **c** and **d** Diffuse Alveolar Damage, intermediate. Alveolar spaces are diffusely filled with oedematous, myxoid connective tissue, indicating areas of early fibroproliferation. In **d**, one can observe the presence of hyaline membranes. **e** Alveolar haemorrhage. The alveolar spaces are filled with red blood cells and oedematous fluid. **f** Bronchopneumonia. Alveolar cells are filled with neutrophils and macrophages. There are no hyaline membranes. **g** Fibrinous, in organization thrombus in a pulmonary artery branch. **h** Multiple fibrin clots in alveolar capillaries (arrow). In the link https://pathpresenter.net/#/public/display?token=5a84d5d9 one has access to the scanned images of the examples above

Histopathological examination was performed by experienced pulmonary pathologists who were unaware of clinical and image patterns, and that quantified the proportion (expressed as percentage) of pulmonary tissue exhibiting the following histological patterns: normal lung, exudative DAD, intermediate DAD, advanced DAD, and acute bronchopneumonia. The histological criteria used were the following: (a) normal lung: lung parenchyma with normal histology or minimal non-specific changes as mild oedema and congestion; (b) exudative DAD: interstitial and/or intra-alveolar oedema, interstitial inflammation, variable amounts of alveolar haemorrhage and fibrin deposition, intra-alveolar hyaline membranes and type II pneumocyte hyperplasia; (c) intermediate DAD: any degree of fibroblastic proliferation within the interstitium and/or alveolar spaces, including loose aggregates of fibroblasts admixed with scattered inflammatory cells, collagen deposition, squamous metaplasia, intermingled with areas with hyaline membranes; (d) advanced DAD: the same as above, with predominance of densely fibrotic areas and no hyaline membranes. The vascular alterations were quantified as the number of pulmonary small arteries with thrombi in the vascular lumen, a binary indicator of the presence of capillary thrombi (0 = absence; 1 = presence), and the proportion of pulmonary tissue with infarction and haemorrhage in each lung site. The diameter from the small, intra-acinar arteries varied from 60 to 250 µm, with very few larger arteries present in the sample [14]. Bronchopneumonia was considered when there was an accumulation of intra-alveolar neutrophils, macrophages, and cell debris within at least 10 contiguous alveolar spaces. Lung haemorrhage was considered when at least 10 contiguous alveolar spaces were filled with blood. The measures of the histological parameters obtained from the different sampling sites were averaged to produce a single estimate for each case, except for the binary indicator of presence of capillary thrombosis, whose aggregated estimate was based on the sum of all the biopsies of the eight lung sites. Figure 1 shows representative figures of the main histopathological parameters considered in the present study.

### Statistical analysis

Data are presented as median and ranges. We assessed the prevalence of each histological pattern along the clinical evolution of disease. For such purposes, each patient was assigned to one of three groups, depending on the time interval between hospital admission and deaths. Such groups were established on the basis of the observed distribution of time evolution of our patients, categorized in tertiles. Comparison among groups was performed using Kruskal–Wallis statistics. To test the co-variation amongst variables, Hierarchical Cluster and Principal Component analysis with Varimax rotation was used. Spearman correlations were performed between clinical-demographic parameters and histopathological patterns. Statistical analyses were performed with the aid of the SPSS V25 software. The level of significance was set in p < 0.05.

## Results

We evaluated 41 patients (18 female and 23 men) with ages varying from 22 to 88 years (median 55 years). The time elapsed from initial symptoms to death varied from 3 to 47 days (median 18 days), the length of hospital stay varied from 0 to 40 days (median 12 days) and intensive care stay varied from 0 to 38 days (median 9 days). All patients had laboratory confirmation of SARS-COV-2 infection by a positive RT-PCR result on the naso/oropharyngeal swab and/or lung tissue. Table 1 shows demographic and clinical characteristics of patients evaluated in this study. Part of this population has been previously presented [3].

**Table 1:** Demographic and clinical characteristics of the 41 patients that died due to COVID-19

Female/male	18/23
Caucasians, n (%)	35 (85%)
Age in years, median (range)	55 (22—88)
Body mass index, median (range)	26 (13—48)
*Comorbidities, n (%)*	
Diabetes	13 (32.5%)
Systemic arterial hypertension	20 (50%)
Cardiomyopathy	10 (25%)
Vascular disase	5 (12.5%)
Chronic kidney disease	4 (10%)
Asthma	2 (5%)
Chronic obstructive pulmonary disease	5 (12.5%)
Smoker	4 (10%)
Comorbidities number	
1	20 (54.1%)
2	10 (27%)
3	2 (5.4%)
4	3 (8.1%)
5	2 (5.4%)
*Symptoms, n (%)*	
Fever	29 (72.5%)
Dyspnoea	33 (82.5%)
Cough	27 (67.5%)
Rhinorrhea	6 (15%)
Diarrhoea	9 (22.5%)
Myalgia	14 (35%)
Sore throat	5 (12.5%)
Nausea and vomiting	10 (25%)
Others	15 (37.5%)
Use of corticosteroids, *n (%)*	23 (57.5%)
Use of anticoagulants, *n (%)*	11 (30.8%)
Use of vasoactive drugs (admission), *n* = *37*	15 (40.5%)
Use of mechanical ventilation, *n* = *37*	37 (100%)
Prone positioning, *n* = *37*	12 (32.4%)
Extracorporeal membrane oxygenation, *n* = *37*	4 (10.8%)
Dialysis, *n* = *37*	20 (54.1%)
Glasgow coma scale (day 1), *n* = *37*	3 (3–15)
Simplified acute physiology score III (day 1), *n* = *37*	66 (39–97)
*Laboratory parameters from 24 h before death, median (range)*	
D Dimer (ng/ml), *n* = *32*	4593 (815–126,078)
Fibrinogen (mg/dl), *n* = *20*	485.5 (89–2465)
Platelets (ml/mm^3^), *n* = *39*	158,000 (4000–322,000)
Activated partial thromboplastin time (s), *n* = *32*	1.23 (0.92–7.0)
Lymphocytes (ml/mm^3^), *n* = *39*	0.37 (0.04–1.05)
*Lung parameters from 24 h before death, median (range)*	
PEEP (cmH_2_O)*, n* = *35*	10 (5–20)
FiO_2_ (%)*, n* = *35*	60 (25–100)
PaO_2_/FiO_2_ ratio, *n* = *28*	122.5 (56–430)
Time from symptom onset to hospitalization in days, median (range)	5 (0–26)
Time from symptom onset to death in days, median (range)	18 (3–47)
Period of hospitalisation in days, median (range)	12 (0–40)
Intensive care unit stay in days, median (range)	9 (0–38)
Period of mechanical ventilation in days, median (range)	11 (0–39)

*PEEP* Positive end-expiratory pressure, *FiO*_*2*_ fraction of inspired oxygen ratio, *PaO*_*2*_*/FiO*_*2*_* ratio* arterial oxygen partial pressure/inspired oxygen fraction ratio

Patient care followed institutional protocols developed specifically for COVID-19 patients. Specific drugs for treating COVID-19 were not recommended but could be used at the discretion of the attending physicians. Corticosteroids were not recommended by institutional protocol during the study period, since the results of the large clinical trials that demonstrated efficacy were available only by late June 2020, but were used by approximately two thirds of patients (Additional file 1: Table S1). Remdesivir and convalescent plasma were not available. Prevention of venous thromboembolism (VTE) as recommended with a prophylactic dose low molecular weight heparin or unfractionated heparin for all patients unless they had a contraindication. Standard prophylactic dose of unfractionated heparin or enoxaparin was the most commonly used regimen (see Additional file 1: Table S2), but approximately 10% of patients received intermediate dosing at ICU admission and 5% eventually received therapeutic anticoagulation.

Parenchymal pathological alterations in the form of exudative diffuse alveolar damage were present in the lungs of all patients, with different degrees of extension. Most of the patients presented mixed histological patterns of DAD, with predominance of the exudative and intermediate patterns in 75.6% and 82.9% of the cases analysed. The fibrotic, advanced pattern was present in 39% of the patients.

Sixteen percent of the patients had signs of acute bronchopneumonia, 14.6% had pulmonary infarctions, 43.9% had lung haemorrhage. Areas of acute fibrinous organizing pneumonia were not identified. Thirty-seven patients (90%) presented pulmonary thrombi, 82.9% presented thrombi in the small intra-acinar branches of the pulmonary artery and 51.2% in the alveolar capillaries. Due to the limitations of the MIA/US, thrombosis in larger vessels were not assessed.

Table 2 shows the median (range) proportion of each histological pattern present in the biopsies of the 41 patients of the study. For the thrombotic phenomena, data are shown as the number of small arteries with luminal thrombus, and the sum of lung sites presenting capillary thrombosis in each patient.

**Table 2:** Percentage of lung involvement by different pathological patterns, number of arteriolar thrombosis and sum of biopsied lung sites presenting capillary thrombosis in lung post-mortem biopsies of 41 COVID-19 patients

	Median (range)
Normal lung (%)	16.88 (0–86)
Exudative DAD (%)	20 (0–92.86)
Intermediate DAD (%)	16.67 (0–98.75)
Advanced DAD (%)	0 (0- 99)
Alveolar hemorrhage (%)	0 (0–32.5)
Infarct (%)	0 (0–10.71)
Bronchopneumonia (%)	0 (0–33.75)
Arteriolar thrombosis (number)^a^	0.75 (0–9.75)
Capillary thrombosis (sum)^b^	1 (0–7)

*DAD* diffuse alveolar damage

^a^Number of vessels with thrombosis in each patient

^b^Sum of biopsied lung sites presenting capillary thrombosis in each patient

**Fig. 2:**
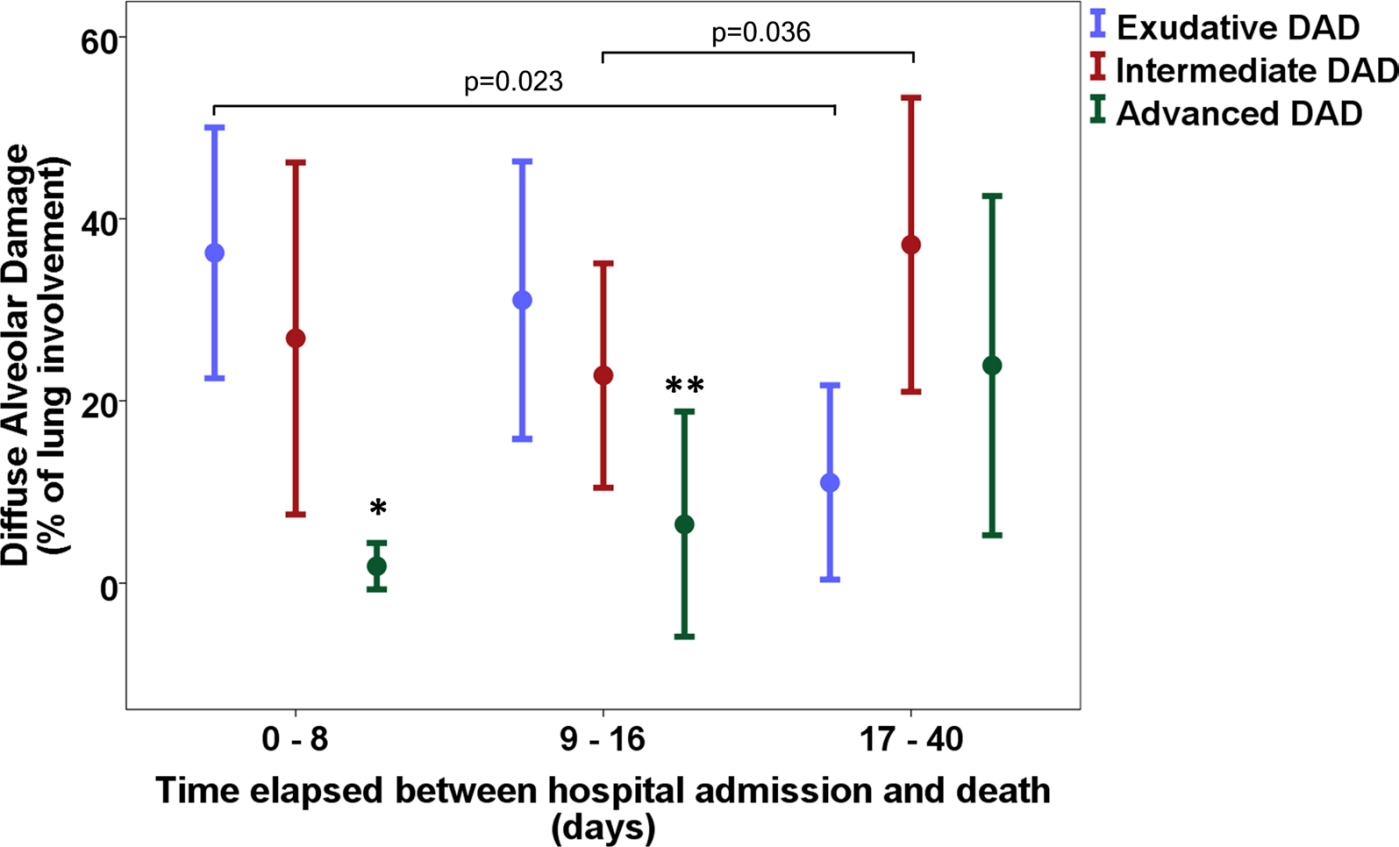
Distribution of the frequency of diffuse alveolar damage patterns in lung biopsies along time of hospital admission until death. 0 to 8 days: *Advanced DAD ≠ exudative DAD (p = 0.005); 9 to 16 days: **Advanced DAD ≠ exudative DAD (p = 0.002) and intermediate DAD (0.014). *DAD* diffuse alveolar damage

The proportion of each histological parameter was tested in the different periods of time elapsed between symptoms onset, hospital admission and intensive care admission till death, ranked in tertiles. Significant results were found when time from hospital admission to death was ranked in tertiles (0 to 8 days; 9 to 16 days; and 17 to 40 days), as seen in Table 3. There were significant differences between exudative DAD (p = 0.017) and advanced DAD (p = 0.036) along the tertiles of time. The proportion of the exudative DAD pattern was significantly higher in the first tertile when compared to the third tertile (p = 0.023). On the other hand, the proportion of the intermediate DAD pattern was statistically higher in the third tertile when compared to the second tertile (p = 0.036), as indicated in Fig. 2. There were no differences in the intermediate DAD pattern over time. Within the time tertiles, there were significant differences between the exudative and advanced pattern in the first tertile (p = 0.005) and between the exudative (p = 0.002) /intermediate patterns (p = 0.014) in the second tertile.

**Table 3:** Frequency of pathological patterns parameters defined by tertiles of time from hospital admission till death (0 to 8 days; 9 to 16 days; and 17 to 40 days). Data presented as median (range)

	Time elapsed between hospital admission and death			*P
	0 to 8 days (n = 13)	9 to 16 days (n = 15)	17 to 40 days (n = 13)	
Normal lung	18.33 (0–86)	15.71 (0–85)	24 (0–83.29)	0.953
Exudative DAD	31.25 (0–75.71)^#^	27.38 (0–92.86)	0.13 (0–58.75)	***0.017***
Intermediate DAD	14.29 (0–98.75)	12.5 (0–77.63)	38.57 (0–90.63)	*0.355*
Advanced DAD	0 (0–16.88)	0 (0–92.86)^†^	2.5 (0–99)	***0.036***
Alveolar Hemorrhage	0 (0–20)	0.14 (0–32.5)	0 (0–8.75)	*0.412*
Infarction	0 (0–0)	0 (0–1.43)	0 (0–10.71)	*0.192*
Bronchopneumonia	0 (0–33.75)	0 (0–15)	0 (0–5)	*0.372*
Arteriolar thrombosis	0.5 (0–1.38)	1 (0–3.67)	0.71 ( 0–9.57)	*0.200*
Capillary thrombosis	0 (0–6)	2 (0–6)	0 ( 0 -7)	*0.473*

*P = Comparison of a given histological parameter among tertiles of time elapsed between admission and death. Exudative DAD: ^#^0 to 8 days ≠ 17 to 40 days (p = 0.023); Advanced DAD: ^†^9 to 16 days ≠ 17 to 40 days (p = 0.036)

**Fig. 3:**
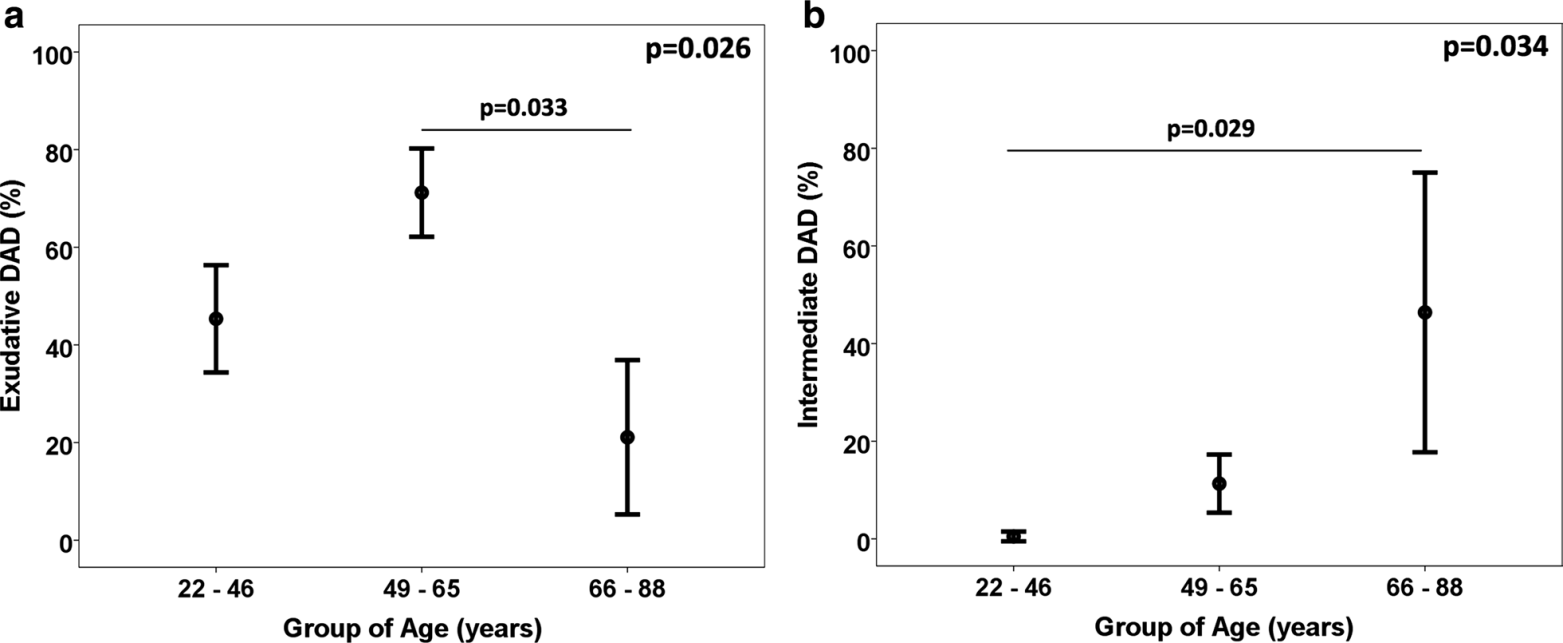
Influence of age on the frequency of diffuse alveolar damage (DAD) patterns in patients that died within the 8 days of hospital admission

There were significant differences among the age groups within tertiles of hospital admission. Older patients (66–88 years) that died within the first eight days of hospital admission presented significantly less proportion of the exudative pattern compared to patients in the age group 49–65 years (p = 0.033), and increased proportion of the intermediate pattern compared to younger patients (22–46 years, p = 0.029) (Fig. 3).

**Fig. 4:**
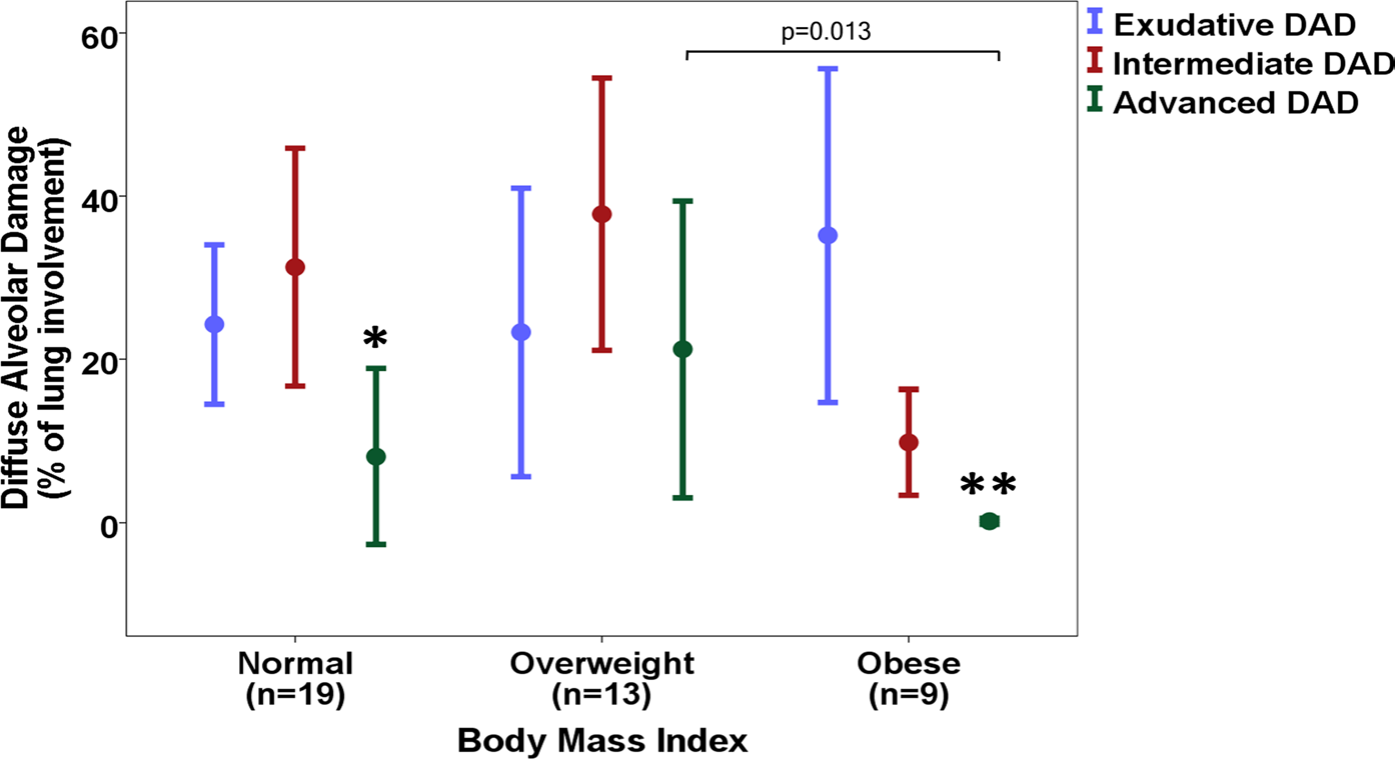
Influence of weight on the frequency of diffuse alveolar damage patterns in the 41 patients that died of COVID-19. Normal: *Advanced DAD ≠ exudative DAD (p = 0.006) and intermediate DAD (p = 0.017); Obese: **Advanced DAD ≠ exudative DAD (p = 0.014). *DAD* diffuse alveolar damage

On the other hand, in obese patients, the frequency of advanced DAD was significantly lower when compared to the exudative DAD (p = 0.014), with a pattern like patients with normal weight. When compared to patients with normal and overweight, obese patients had lower proportion of the advanced pattern (p = 0.013), Fig. 4. The relatively small number of obese patients did not allow further analysis in tertiles of hospital admission days. For all other histological parameters analysed, including the percentage of normal lung in biopsies, there were no differences in the proportion of patterns over time of hospital admission or demographic/clinical parameters.

**Fig. 5:**
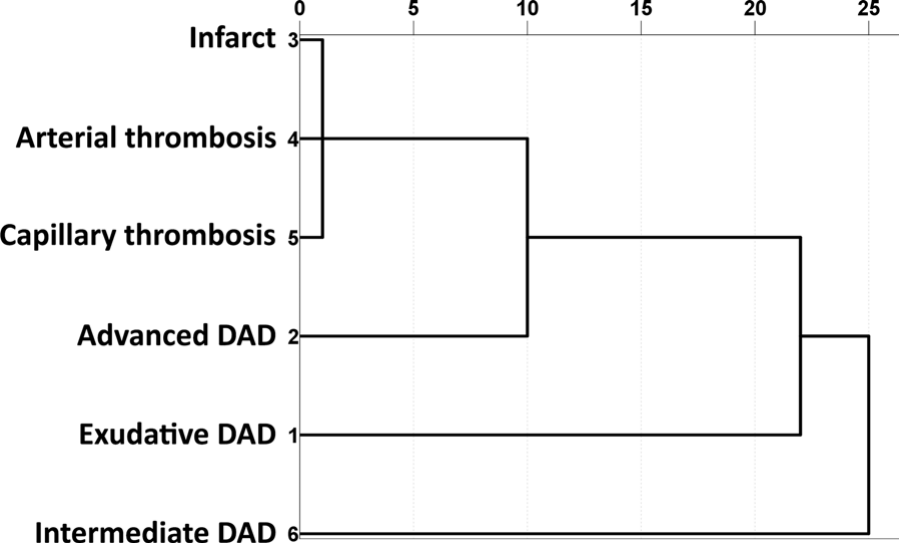
Dendrogram showing that estimators of vascular pathology aggregated in the same cluster, but not the diffuse alveolar damage (DAD) patterns

Next, we performed Hierarchical Clustering Analysis to check whether there was any covariation of the histopathological patterns that could be linked by similar pathogenetic mechanisms, for instance. As can be depicted in Fig. 5, the estimators of vascular pathology aggregated in the same cluster, but not the DAD patterns. We further explored this aspect with an exploratory Principal Component Analysis (PCA), using a rotated solution (Varimax) to identify how histopathological patterns varied together. Variables with a factor loading score of 0.7 were used to assist in the interpretation of rotated factors followed by PCA. Factor 1 was related to normal lung and intermediate DAD, factor 2 was associated with the exudative and advanced DAD, and factor 3 was related to arterial thrombosis, showing that vascular changes clustered apart from DAD patterns. The component’s matrix is presented in Table 4.

**Table 4:** Principal component analysis (PCA), using a rotated solution (Varimax), with the diffuse alveolar damage and vascular parameters

	Principal component analysis		
	1	2	3
Normal lung	− 0.925	− 0.054	0.206
Exudative DAD	0.078	− 0.743	− 0.449
Intermediate DAD	0.732	0.028	0.384
Advanced DAD	0.107	0.882	− 0.186
Arterial thrombosis	− 0.087	0.119	0.713
Capillary thrombosis	0.368	− 0.146	0.585

Factor 1 was related to normal lung and intermediate DAD, factor 2 was associated with the exudative and advanced DAD, and factor 3 was related to arterial thrombosis, showing that vascular changes clustered apart from DAD patterns

*DAD* diffuse alveolar damage. Variables with a factor loading score of 0.7 were used to assist in the interpretation of rotated factors followed by PCA

There were no correlations among the histological patterns and use of corticosteroids or anticoagulants. As expected, there were negative correlations between exudative DAD and period of hospitalization (r = − 0.419, p = 0.006), stay in intensive care units (r = − 0.507, p = 0.001) and days in mechanical ventilation (r = − 0.426, p = 0.007). Of importance, there were no correlations between age and BMI with the length of stay in the hospital/ the ICU or the interval between hospital admission and death.

## Discussion

In this study, we analysed, in a semiquantitative manner, the different and concomitant histopathological patterns of lung involvement in patients that died due to COVID-19, stratified by different periods of disease involvement. Our results show that patients with severe fatal COVID-19 presented a mixed spectrum of different proportions and patterns of DAD along different periods of hospital stay, with an increase in the proportion of the more advanced/fibrotic pattern in patients that had longer periods of hospitalization. Interestingly, older patients that died within shorter periods of hospital stay (8 days) had a significantly larger proportion of the intermediate, fibroproliferative pattern and less of the exudative pattern than the younger patients that died in the same time frame. Obese patients, on the other hand, had a lower proportion of the advanced pattern in their biopsies. No other associations were found for all other histological parameters, including the very frequent finding of pulmonary microthrombosis, which is present in a large proportion of patients, regardless of the duration of the disease. Our attempt to identify clusters of histopathological patterns showed that the vascular patterns aggregated differently from the DAD patterns.

Our data confirmed previous studies addressing this subject. Polak et al. performed a systematic review of COVID-19 autopsies, and identified that vascular injury and acute DAD occur along all the time frames of disease evolution, but fibrotic DAD tends to occur after three weeks [9]. Li et al. demonstrated that patients with fibrosing DAD, compared to those with acute DAD, were one decade younger and had significantly longer duration of illness, hospitalization, and mechanical ventilation [5]. Our group of younger patients were younger than the patients presented by Li et al., which might help explain the discrepancy between their and our findings. In addition, in our study, older patients presented earlier (up to 8 days of hospital admission) fibroproliferative responses than younger patients, but we found no differences in the later periods of hospital admission. It is also possible that, in Li´s et al. study, the younger adults that survived more days in the hospital developed more fibrotic lesions in response to DAD than the older subjects that had died before. In our study, we tested this hypothesis, that was not the case.

The extent and severity of pulmonary complications in COVID-19 have not been fully determined, but many patients persist with respiratory symptoms in the aftermath. Huang et al. reported that a third of recovered patients presented fibrotic alterations at discharge [15], suggesting a future burden of chronic lung disease post COVID-19 pandemic. The most common abnormality of lung function in patients with COVID-19 discharged from the ICU is impairment of diffusion capacity, followed by restrictive ventilatory defects, which are both associated with the severity of the disease [16]. DAD represents a time-dependent, stereotypic response of the lungs to severe injury of different etiologies, with the development of fibrosis after three weeks of mechanical ventilation in 60% of the cases [17]. It has still to be determined whether COVID-19 will lead to a higher frequency of late pulmonary complications when compared to other causes of ARDS, and whether specific treatments and ventilation modes could change disease evolution. So far, Konopka et al. in a study with few cases, could not identify differences in DAD patterns in patients dying outside or inside the hospital setting, or when compared with lung tissue of patients that died of other causes of ARDS [18]. The presence of acute lesions intermingled with fibroproliferative DAD in COVID-19 suggests room for improvement with protective ventilation strategies and anti-fibrotic therapies in this disease.

We and others have shown a high frequency of vascular events in the lungs of critical COVID-19 patients [2, 3, 9, 12] that are present early in the course and along all stages of the disease, apparently occurring “time independently” of the different DAD patterns. Vascular pathology is very common in ARDS of different causes, varying from ultrastructural changes in the endothelium, thrombotic events due to altered coagulation mechanisms, and vessel wall remodelling in later stages. Tomashefski et al. performed a dedicated study to identify vascular changes in ARDS and found vascular alterations in 95% of the cases [19]. On the other hand, the demonstration of coronavirus particles in endothelial cells of COVID-19 autopsies indicates that direct microvascular injury plays a mechanistic role in the more severe cases of the disease. Further, it is possible that pre-existing comorbidities predispose these patients to endothelial fragility. As suggested by Wichmann et al., it is possible that all these factors in fact act in a synergistic manner, explaining the high frequency of thrombotic events in COVID-19 [20]. Indeed, Ackerman et al. demonstrated that microthrombi were nine times as prevalent in patients that died due to COVID-19 as in patients that died due to influenza infection [11].

Age is the most important risk factor for disease severity and mortality in COVID-19 [21]. In our series, older patients that died within the first 8 days of hospital admission had a larger proportion of the fibroproliferative and less of the exudative patterns in their lung samples. It is still unclear whether pulmonary reparative responses linked to aging, upon other senile related frailties, have a role in COVID-19 adverse outcomes. There is overall little information on the mechanisms involved in lung repair in aging. Unbiased microarray analysis of normal lungs revealed that age-related increases in collagen were higher in elderly patients compared with those in the third and fourth decade of life [22]. In aged rodents, we have previously shown increases in the levels of lung collagen [23]. Our data suggest that responses in the elderly after acute lung injury might involve earlier fibrogenesis. Accordingly, Wei et al. analysing thin lung CT scans of COVID-19 patients after hospital discharge observed that older patients with more severe forms of the disease were more prone to develop lung fibrosis [24].

Conversely, we identified that in obese patients, there was a predominance of exudative upon the fibrotic patterns in lung biopsies. Obesity is a compound risk factor for severe COVID-19 infection [25], but there is little data on the influence of obesity in the pathology of ARDS/COVID-19. Maia et al. demonstrated that obese mice with experimentally induced lung injury develop less signs of lung remodelling, such as lower levels of TGF-beta and collagen when compared to non-obese mice [26]. The Obesity-ARDS paradox refers to the fact that although obesity is associated with an increased risk of ARDS, obese patients had a lower mortality risk when compared to patients with a normal BMI in some studies [27]. There is ongoing discussion whether this paradox has been broken by COVID-19, but our data suggest indeed a difference in response to injury in obese patients [28].

This study has several limitations. We have not compared COVID-19 cases with ARDS of different etiologies, to detect potential alterations particular to the disease and not a stereotyped response to ARDS/ventilation. We have worked with post-mortem biopsies, making it more difficult to foresee a more complete histological picture. On the other hand, biopsies represented eight sites of the lung, allowing to adequately map disease distribution. Although the relatively small number of patients did not allow us to make robust associations with clinical parameters or ventilatory patterns that could have had influence on injury patterns, we were able to identify associations with parameters such as age and weight that deserve to be explored in larger cohorts.

In summary, patients with severe COVID-19 present different proportions of acute and fibroproliferative DAD patterns along the disease course, with the fibrosing pattern being more prevalent after 17 days of hospital admission. These patterns seem to be influenced by age and weight. Vascular microthrombotic changes are extremely frequent, do not show any temporal distribution, cluster apart from the diffuse alveolar damage patterns, and are not influenced by individual factors. These data may be helpful to better understand disease evolution and outcomes, such as lung fibrosis post-COVID-19.

## Supplementary Information

**Additional file 1: Table S1.** Use of corticosteroids during ICU stay (n=41). **Table S2:** Anticoagulation therapy used during ICU stay (n=37).

## Data Availability

For access to the research material and other information related to COVID-19 autopsies, contact tmauad@usp.br.

## Abbreviations

ARDS: Acute respiratory distress syndrome
BMI: Body mass index
COVID-19: Coronavirus disease 2019
DAD: Diffuse alveolar damage
H&E: Haematoxylin and eosin
MIA/US: Ultrasound-guided minimally invasive autopsies
PCA: Principal component analysis
RT-PCR: Reverse transcription polymerase chain reaction
SARS-CoV-2: Severe acute respiratory syndrome coronavirus 2
TGF-beta: Transforming growth factor beta
