# Supplementary material for: Tracking the time course of pathological patterns of lung injury in severe COVID-19
Source: Respir Res. 2021 Jan 29;22:32. doi: 10.1186/s12931-021-01628-9 (PMC7844838; doi:10.1186/s12931-021-01628-9)
Supplement: Supplementary file 1 — Additional file 1: Table S1. Use of corticosteroids during ICU stay (n=41). Table S2: Anticoagulation therapy used during ICU stay (n=37). [file 12931_2021_1628_MOESM1_ESM.docx]

**Tracking the time course of pathological patterns of lung injury in severe COVID-19**

Thais Mauad^1^_,_ Amaro Nunes Duarte-Neto^1^, Luiz Fernando Ferraz da Silva^1,2^_,_ Ellen Pierre de Oliveira^3^, Jose Mara de Brito^1^, Ellen Caroline Toledo do Nascimento^1^, Renata Aparecida de Almeida Monteiro^1^, Juliana Carvalho Ferreira^3^, Carlos Roberto Ribeiro de Carvalho^3^, Paulo Hilário do Nascimento Saldiva^1^, Marisa Dolhnikoff^1^

1. Departamento de Patologia, Faculdade de Medicina da Universidade de São Paulo.

2. Serviço de Verificação de Óbitos da Capital, Universidade de São Paulo.

3. Departamento de Cardiopneumologia, Instituto do Coração, Faculdade de Medicina da Universidade de São Paulo.

**Corresponding Author:**

Prof. Dr. Thais Mauad

Faculdade de Medicina da Universidade de São Paulo, Departamento de Patologia Av. Dr. Arnaldo, 455, sala 1155 - Cerqueira Cesar, São Paulo - SP, 01246-903 Phone number: 55+11+3061-7173,

e-mail: tmauad@usp.br

**Additional Information**

**RESULTS**

**Additional Table S1:** Use of corticosteroids during ICU stay (n=41).

| **Drug and dose** | **Number of patients** | **Duration of use, days** |
| --- | --- | --- |
| Hydrocortisone 50mg 6/6 hours | 14 (34%) | 3 (2-5) |
| Hydrocortisone 100mg 8/8 hours | 4 (10%) | 6 (1-11) |
| Methylprednisolone - up to 1mg/kg | 4 (5%) | 10 (9-13) |
| Methylprednisolone ≥ 2mg/kg | 2 (5%) | 6.30* |
| Dexamethasone 10mg/day | 1 (2%) | 9^#^ |
| Prednisone 40mg | 2 (5%) | 3.4* |
| None | 14 (34%) | NA |

**Legend:** mg: miligrams; kg: kilograms; NA: not applicable. Data are expressed as median (25-75% IQR), unless otherwise stated; *data expressed as range; data expressed as the number of days the only patient receiving this dose received.

**Additional Table S2:** Anticoagulation therapy used during ICU stay (n=37).

|  | **First 24 hours** | **48 hours before death** | **24 hours before death** |
| --- | --- | --- | --- |
| Prophylactic standard dosing |  |  |  |
| Heparin 5000 UI - 8/8 hours | 9 (24%) | 9 (24%) | 13 (35%) |
| Heparin 5000 UI - 12/12 hours | 3 (8%) | 3 (8%) | 4 (11%) |
| Enoxaparin 40 mg - 1x/day | 14 (38%) | 6 (16%) | 2 (5%) |
| Prophylactic intermediate dosing |  |  |  |
| Heparin 5000 UI - 6/6 hours | 1 (3%) | 2 (5%) | 2 (5%) |
| Enoxaparin 60 mg - 1x/day | 2 (5%) | 2 (5%) | 1 (3%) |
| Enoxaparin 40 mg - 12/12 hours | 2 (5%) | 4 (11%) | 2 (5%) |
| Therapeutic anticoagulation |  |  |  |
| Continuous unfractionated heparin | 2 (5%) | 5 (14%) | 5 (14%) |
| Enoxaparin 1mg/kg - 1x/day | 0 | 20 | 1 |
| None (contraindication) | 4 (11%) | 6 (16%) | 8 (22%) |

**Legend:** UI: international units; mg: milligrams; data on anticoagulation was missing for 4 patients.
